# Solid-State NMR Spectroscopy Investigation of Structural Changes of Mechanically Strained Mouse Tail Tendons

**DOI:** 10.1021/jacs.4c13930

**Published:** 2025-03-08

**Authors:** Thomas Kress, Melinda J. Duer

**Affiliations:** Yusuf Hamied Department of Chemistry, University of Cambridge, Cambridge CB2 1EW, United Kingdom

## Abstract

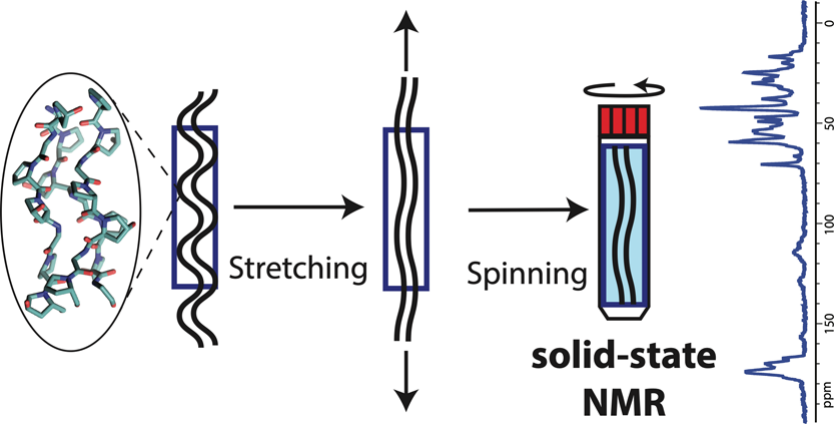

Structural tissues like tendon are subjected to repeated tensile strains *in vivo* and excessive strains cause irreversible changes to the tissue. Large strains affect the molecular structure and organization of the extracellular matrix, and these are the parameters that drive cell behavior, including tissue repair. Here we describe a method to perform solid-state NMR spectroscopy on *in situ* strained tissue samples under magic-angle spinning to achieve high-resolution NMR spectra while maintaining the tissue’s native hydration state. The changes observed in the NMR spectra are interpreted using quantum mechanics molecular mechanics (QM/MM) chemical shift calculations on strained collagen triple-helix structures and consideration of changes in the distribution of molecular orientations between strained and relaxed mechanical states. We demonstrate that our tissue strain method in combination with spectral simulations can detect changes in collagen organization between tendons loaded to plastic deformation and subsequent structural relaxation in the unloaded state.

## Introduction

What happens to the molecular structure and dynamics of our structural tissues as they are stretched and released is an important question in biomaterials and for tissue engineering. Structural tissues like tendon contain a collagen-rich extracellular matrix (ECM) that bestows the unique mechanical properties of each tissue. Equally importantly, the molecular structure and three-dimensional (3D) molecular organization of the ECM is key in determining the physiology of the cells in the tissue through the numerous, highly dynamic interactions between ECM biopolymers and the cell surface receptors. Thus, changes in ECM structure or organization through mechanical processes are expected to have a major impact on cell behavior, and hence tissue physiology, as well as on the tissue’s mechanical properties.

The predominant ECM biopolymers are collagens, triple helical proteins that assemble into fibrils, fifty to several hundred nanometers in diameter. Collagen molecules are organized in highly ordered arrays within the fibrils, the molecular organization stabilized by intermolecular cross-links and charge–charge interactions between residues on neighboring collagen molecules.^1−3^ Electron spin resonance (ESR) studies^4,5^ showed that straining rat tail tendons into the plastic deformation region caused the production of radicals,^4^ hypothesized to be due to the breakage of intermolecular collagen cross-links. Another study^6^ discovered that the density of collagen cross-links was significantly lower in tendons strained to plastic deformation compared to unstrained controls, but that the cross-link density recovered in part upon relaxation of the mechanical strain. These studies^4,6,3^ imply that there is considerable scope for reorganization of molecules in the ECM through plastic deformation and subsequent structural relaxation. Strain into the plastic deformation regime takes the tissue beyond its elastic strain limit, and so subsequent structural relaxation cannot return the molecular structure and organization to the original ECM structure. Rather, some permanent structural deformation persists. One of the open questions is what happens to the collagen molecular organization and structure after release of the stress that created a plastic deformation? Such a chemical state is likely to be common in tendon injuries and may be manifest in other scenarios, for instance as a result of local mechanical strain/release induced by migrating cancer cells in a tumor setting.^7,8^ The chemical environment that cells detect after relaxation of the strain that takes the extracellular matrix into a damaged regime is fundamental in determining how cells then respond to the damage. There are however currently few methods that allow *in situ* investigation of molecular structure changes in such a complex material as the extracellular matrix^9,10^ and even fewer that allow study while the material is held under mechanical strain. Here, we demonstrate that solid-state NMR spectroscopy in combination with spectral simulations can be used to compare the collagen molecular conformation and organization in tendons held strained in the plastic-deformation region and the same tendons after subsequent release of the mechanical strain.

Solid-state NMR spectroscopy is potentially an excellent method to probe the molecular processes that occur as mechanically strained tissues relax. Previous work^11−17^ has shown that the dominant ^13^C and ^15^N NMR signals from intact solid tissues such as tendons can be assigned to collagen and moreover that chemistry occurring on collagen molecules in tissues can be detected by solid-state NMR.^18−20^ Spinning the sample at the magic-angle at kilohertz frequencies, so-called magic-angle spinning (MAS), is however a prerequisite for obtaining high-resolution spectra of solid samples in NMR. This represents a challenge for *in situ* study of materials under mechanical strain. We address this challenge in this work and present a method to perform MAS NMR experiments on strained, intact biological tissue, that also allows relaxation of the strain when required. Observed spectral changes are then analyzed using quantum mechanics molecular mechanics (QM/MM) chemical shift calculations on strained collagen triple-helix structures and by considering possible changes in the collagen molecular orientation distribution between the strained and relaxed states.

*In situ* sample stretching in NMR (without magic angle spinning) was first applied to study the mechanical relaxation properties of aluminum metal sheets,^21^ which then inspired other *in situ* studies further developing *in situ* straining,^22^ and studies on polymer conformational changes^23−25^ or polymers exogenously strained to induce plastic deformation.^25,26^ These latter studies followed ^13^C chemical shift anisotropy and changes of chemical shifts but used static (non-MAS) sample conditions and so were limited by the spectra resolution available in the resulting spectra.

We have found only a few instances of *in situ* stretching under MAS conditions. Mechanical strain on ^13^C-enriched spider silk fibers was maintained for MAS NMR by drying *ex situ* strained silk fibers while the fibers were held strained before placing the dried silk fibers in an NMR rotor (8.5 kHz MAS rate).^27,28^ Schmidt et al. performed the material stretching step on a rectilinear polymer block *ex situ*,^29^^29^ and maintained the polymer block in its stretched state by a collar clamped around the polymer block that was then fitted tightly inside a 7 mm rotor; this construct was successfully spun at 4 kHz MAS rate. Kimura et al. stretched polymers rings around a cylinder that fit inside a 7 mm rotor,^30^ which allowed MAS NMR spectroscopy on the polymer rings at MAS rates up to 5 kHz. These latter two approaches are well-suited for studying polymers that can be fabricated into specific sample geometries with a high degree of precision, and both led to intriguing insight into polymer molecular mobility and structure in the strained condition.

However, none of the methods mentioned above are suitable for intact mammalian tissues where the aim is to gain insight into the *in vivo* tissue environment. Native mammalian tissues are highly hydrated, and their hydration state must be maintained for results to have relevance to *in vivo* tissues. Mammalian tissues typically have heterogeneous geometries and are not readily fabricated into alternative sample geometries without compromising tissue integrity and introducing unintended variability between samples. Mammalian tissues also have considerable compositional complexity and require >7 kHz MAS to give resolution in ^13^C NMR spectra that avoids spinning sidebands overlapping with other signals. Finally, the experimental setup must allow for potentially long NMR acquisition times, as the high hydration level of most biological tissues means that the concentration of the biomolecule of interest is relatively low compared to typical synthetic solid polymer samples, for instance.

## Results and Discussion

**Figure 1:**
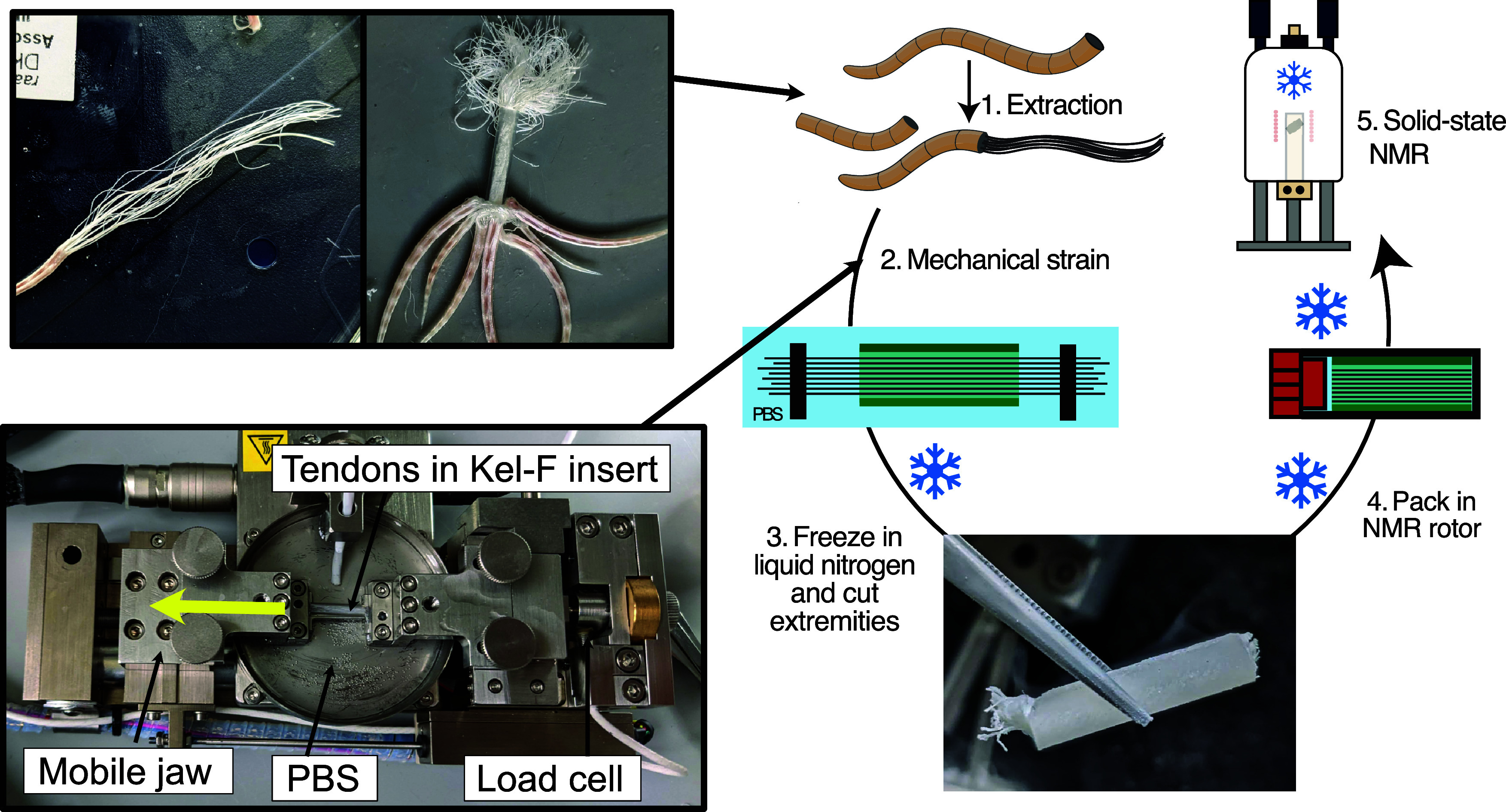
Method for performing MAS NMR on mechanically strained tissue samples. Tendons are maintained in PBS to maintain the native tissue hydration throughout the process. (1) Tendons from 7 mouse tails are threaded through a Kel-F tube. (2) Tendons are mounted in a straining device, strained to plastic deformation while recording stress–strain curves. (3) Tendons are flash-frozen in liquid nitrogen, and their ends are trimmed with a scalpel. (4) The Kel-F insert is packed into a liquid nitrogen-cooled 4 mm rotor. (5) The rotor is loaded into a precooled 4 mm probe for MAS NMR analysis.

The method we developed for performing high-resolution MAS NMR spectroscopy on tensile-strained biological tissues is outlined in Figure 1A. It takes inspiration from the work of Schmidt et al.,^29^ who used a close-fitting collar around an ex situ-strained sample to hold the strain in place. In our case, the collar is ice, formed from the buffer that we immerse the tissue sample in to maintain its hydration during the mechanical straining. In our work here, the biological tissue of interest is tendon. Tendon is 70–80 dry wt % collagen and one of the most homogeneous tissues in terms of chemical composition, microarchitecture and mechanical properties, making it an ideal tissue for testing the robustness of our methodology as well as investigating the effect of macroscopic plastic deformation on collagen structure and organization.^3,31^ Furthermore, collagen fibrils are largely aligned with the tendon long axis.^3,31,32^ Mechanical strain can therefore be expected to result in relatively uniform local strain and molecular effects. In this work, we used mouse tail tendons. The distribution of a mechanical strain applied macroscopically in a material depends on the shape and dimensions of the material sample and mouse tail tendons are highly uniform in diameter, so no further sample preparation is required after tendon dissection to generate uniform tissue sample dimensions. Typically, around 200–300 tail tendons from three different mouse tails were threaded through an open-ended Kel-F cylinder (rotor insert that is NMR-invisible in ^13^C cross polarization (CP) spectra used in this work) so that the tendons protruded from either end of the cylinder (Figure 1). The ends of the tendons were then clamped into the jaws of a bespoke-designed, computer-controlled tensile stage and the tendons taken through the required mechanical strain protocol. Tendon hydration was maintained throughout by keeping the tail and extracted tendons immersed in phosphate-buffered saline (PBS). While the tendons were held in their strained state, the PBS bath was rapidly removed, and the sample immediately flooded with liquid nitrogen. This freezes a tightly fitting collar of ice around the sample inside the Kel-F cylinder, preventing release of the strain on the tendons. The ends of the now frozen tendons were then trimmed flush with the cylinder ends and the whole construct placed rapidly into a precooled NMR rotor.

To ensure that the ice collar remained frozen throughout the process, the rotor plus tendon-ice construct was transported on dry ice and immediately spun with cold bearing gas after insertion in a precooled probe. Structural relaxation, which requires substantial side chain and backbone molecular motion, is expected to be absent at the temperature of the experiment (−35 °C) due to the significantly restricted amplitude of collagen backbone motion.^33^ To confirm the absence of significant molecular motion at −35 °C, we measured ^13^C T_1_ relaxation rates (Figure S1). The collagen carbonyl exhibited R_1_(^13^C) ∼ 0.02 s^–1^, and side chain showed R_1_(^13^C) ∼ 0.1 s^–1^. These values are consistent with an essentially immobile protein, where T_1_ relaxation is dominated by reorienting water molecules,^34^ which confirmed that collagen molecular motion was insufficient to permit structural relaxation under our experimental conditions. The presence of mobile water was further checked with ^1^H NMR spectra (Figure S5) which did not display sharp peaks, suggesting that the water remained frozen during the experiment. Our experience is that our protocol results in samples that are readily spun up to at least 9 kHz MAS frequencies at −35 °C, and reproducible NMR spectra over many samples. The strain on the frozen tissue can be released when required by raising the temperature of the sample sufficient to melt the ice collar around the tissue, then refreezing the sample for further NMR study.

**Figure 2:**
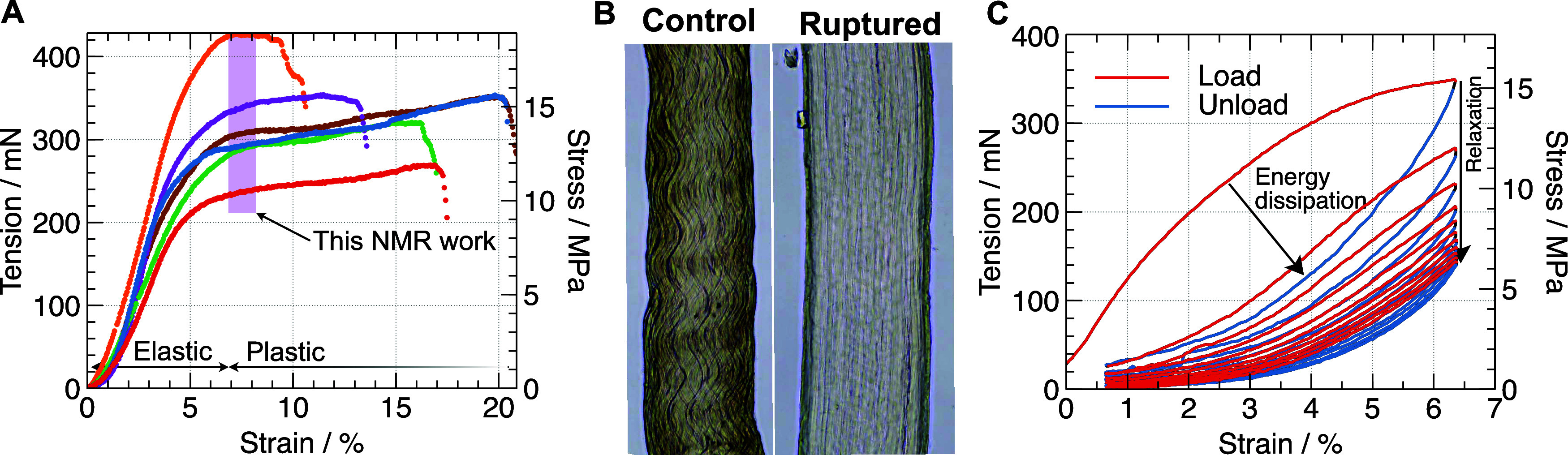
(A) Repeats of strain–stress curves of a single tendon strain at a rate of 1 mm·min^–1^. (B) Phase contrast microscopy images showing permanent damage on a mouse tendon before; and after bringing it beyond its rupture point. Tendons have ca. 170 μm diameter. (C) Experimental stress–strain curves with cyclic loading/unloading cycles. The unloading curves always display lower strains than the loading curve, showing the elastic hysteresis effect typical of viscoelastic behavior.

We then used this method to answer the question: how does the collagen molecular structure and organization change between the plastic deformation strain state and the unloaded state that occurs after release of the stress? In the elastic region of the stress–strain curve (<5% strain, Figure 2A), tendon extension occurs through reversible processes, expected to be a combination of extension and sliding of the collagen units at every level of the tendon hierarchical structure.^35^ Increasing strain beyond the elastic region to the plastic deformation region (>5% strain) causes irreversible structural changes (Figure 2B), where the original tendon structure cannot be obtained by releasing the strain. Repeated loading–unloading cycles into this strain regime (Figure 2C) show typical plastic viscoelastic behavior^3,36^ in which the unloaded (low strain) state on successive load-unload cycles is associated with decreasing tension in the tendon. Several studies^4,6^ have concluded that the intermolecular collagen cross-links break as tensile strain on tendon is increased from the elastic to the plastic deformation region.^6^ We reasoned that when cross-links are broken, collagen triple helices slide further relative to each other, moving out of the local potential energy wells that maintain their ordered state within collagen fibrils (and fibril elasticity),^37^ leading to the possibility of a significantly altered molecular organization upon release of the mechanical strain and subsequent structural relaxation. Therefore, to investigate organizational changes between the plastic deformation strain state and the unloaded, relaxed state, we rapidly strained tendons (2 mm min^–1^) to induce plastic deformation (ca. 7% strain, *i.e.*, ca. 15 MPa achieved in 30s) while minimizing viscoelastic relaxation, characterized them with NMR, and then recorded control spectra by leaving the rotor containing the tendons for 3 days at 4 °C to allow mechanical relaxation in the unloaded state.

**Figure 3:**
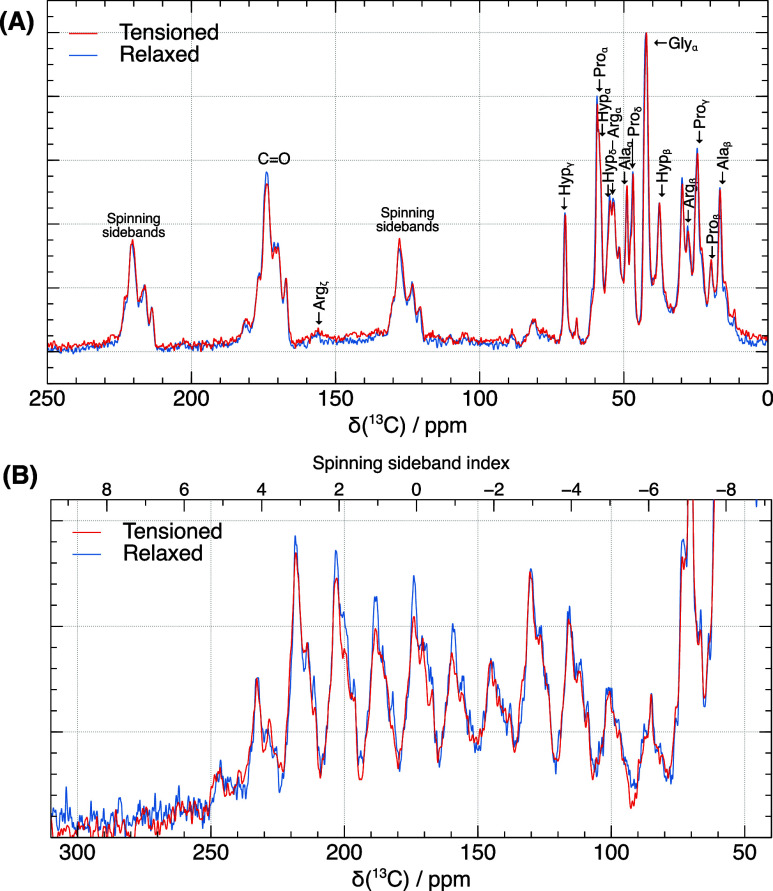
^13^C Cross-Polarization (CP) spectra of mouse tail tendons at 14.1T, −35 °C: (A) 9 kHz MAS rate, highlighting the influence of strain and strain relaxation on NMR spectra. Notably, this effect is evident at lower MAS rate in (B) representing the intensities of the carbonyl spinning sidebands pattern at 2.2 kHz when the spectra are normalized on the intensity of the spinning sideband (index = −3) which highlights the differences observed. (Figure S2 for the full spectra normalized on Gly Cα, and isotropic carbonyl spinning sideband).

Figure 3A shows the ^1^H to ^13^C cross-polarization (CP) spectrum of tendons maintained under tension in the plastic deformation regime (loaded) compared to that recorded after the same tendons were left to relax their structure (unloaded) for 3 days at 4 °C. Cross-polarization was used here to enhance the ^13^C NMR signal, allowing for a high-quality spectrum to be obtained in a few hours. The ^13^C CP spectra of the tendons can be assigned according to the more abundant collagen residues as has been done previously for *in situ* tissue samples:^11,14,13,12,15,17^ Gly (33%), Pro/Hyp (21%), Ala (10%), Arg (5%), Glu (4.5%), Ser (4%), Lys/Hyl (3.5%). Spectra of the strained and relaxed tendons are remarkably similar in the spectral region for α-carbon and aliphatic side chain signals (15–75 ppm) suggesting that there is no significant reorganization of the collagen side chains upon release of strain in the plastic deformation regime. However, the intensity of the carbonyl spinning sidebands changed relative to the carbonyl isotropic signals upon strain release and structural relaxation (Figure 3A). These changes in the spinning sideband distribution suggest alterations in the shielding tensor distribution. This effect is more strongly evident in Figure 3B, where slower MAS, resulting in a more pronounced spinning sideband pattern, was employed.

The ^13^C spectra are from the same tendon sample so that the individual tendon compositions and tendon orientations giving rise to the spectra are highly similar for the strained and relaxed cases. Any differences between the spectra therefore arise from structural changes from relaxation of the mechanical strain. Within tendons, collagen fibrils have preferential alignment with the tendon long axis.^3,31,32^ Thus, we expect preferential alignment of collagen molecules with the rotor axis. Changes in the alignment of collagen molecules after the mechanical strain is released will affect the carbonyl C’ shielding tensor orientations in the rotor and thus the collagen peptide carbonyl spinning sideband intensity patterns. Changes in collagen molecular structure or conformation upon strain relaxation can also be expected to affect the carbonyl ^13^C shielding tensor anisotropy and asymmetry, and potentially the shielding tensor orientations relative to the molecular axis frame too. Thus, we next simulated the trends expected in collagen carbonyl spinning sideband patterns for both changes in distribution of molecular orientations, and for changes in molecular conformation.

### Solid-State NMR Simulations of Spectra of Partially Oriented Tissue Samples

**Figure 4:**
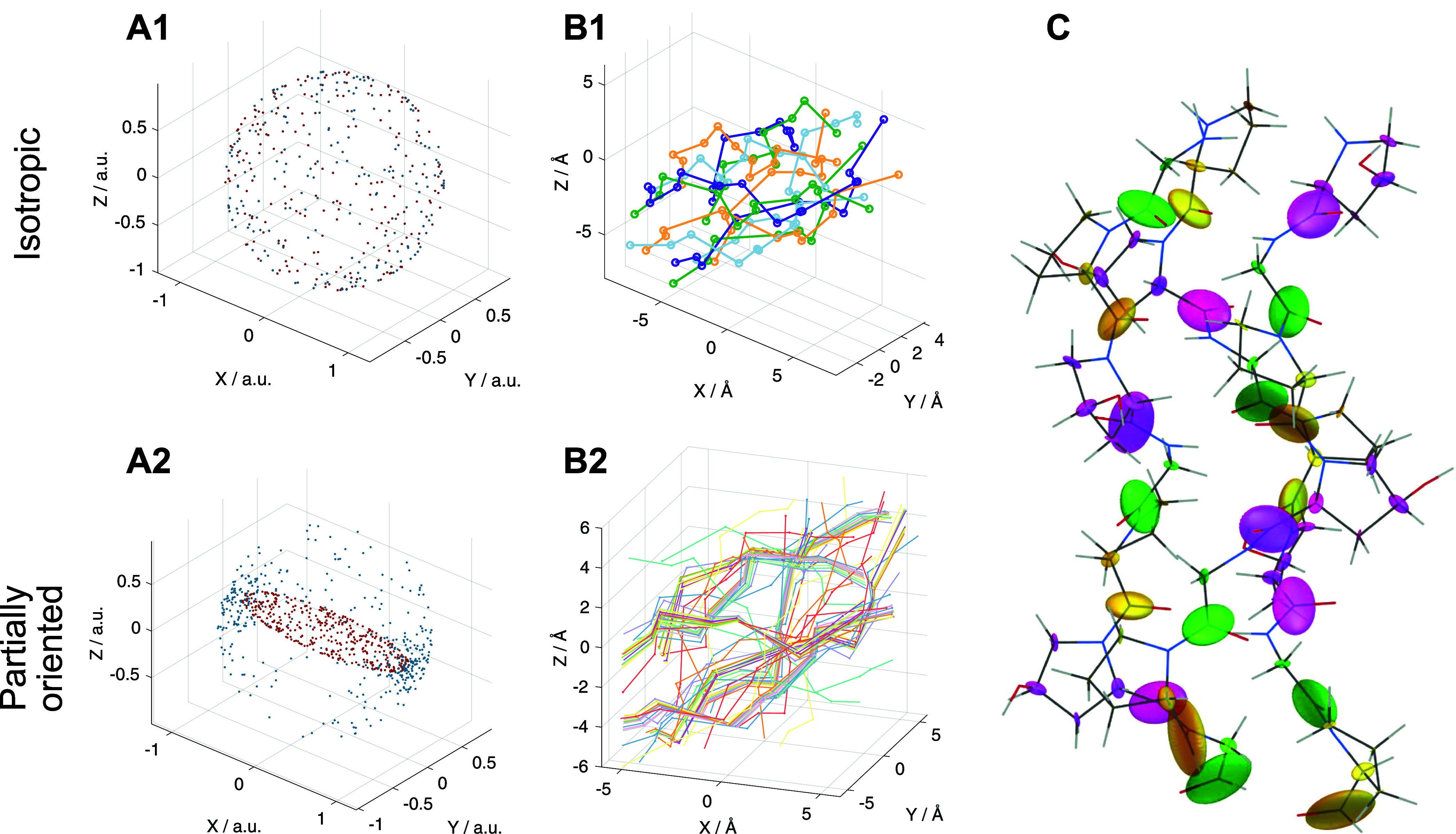
(A) Schematics illustrating how the molecular orientation distribution is calculated for uniform and partially aligned distributions. In the uniform distribution case, random molecular orientation distribution is represented by set of the Euler angles for points randomly distributed on a sphere. Where there are molecules with preferential alignment, the molecular orientation distribution is represented by the Euler angles for uniform point distribution on an ellipsoid projected onto a sphere. Each data point in both scenarios represents a distinct set of Euler angles defining the orientation of the shielding tensors for collagen triple helix backbone carbonyl ^13^C (B) resulting in either (B1) an isotropic distribution of carbonyls or (B2) alignment at the magic angle. (C) Representation of the backbone carbonyl ^13^C shielding tensors calculated for (GPO)_10_. The orientation of each ellipsoid represents the shielding tensor principal axis frame orientation with respect to the molecular frame; the ellipsoid principal axis lengths are proportional to the shielding tensor principal values. Glycine C’ are denoted in green, proline in yellow, and hydroxyproline in magenta.

Simulation of NMR spectra for solid-state samples requires a representative set of molecular orientations describing the statistical distribution of expected molecular orientations in the sample. For a uniform distribution of molecular orientations with respect to the rotor axis frame, *i.e.*, no preferred orientation, a common approach for generating a set of representative molecular orientations is to distribute points uniformly on a sphere, as depicted in Figure 4A1. Each point is associated with Euler angles which are used to define a molecular orientation in the sample with respect to the rotor axis frame (Figure 4B).

To create a set of molecular orientations for a partially aligned sample, we uniformly distributed points on an ellipsoid instead of a sphere and then projected the points onto a sphere to determine the set of Euler angles representing the molecular orientation distribution (Figure 4A2). The long axis of the ellipsoid, denoted as c, was set to be aligned with the rotor long axis. The short axes of the ellipsoid, *a* and *b* are set equal in length here to reflect the expected axial symmetry of tendon alignment in the NMR rotor. The ratio *c*/*a* determines the degree of molecular alignment with the rotor axis; *c*/*a* = 1 represents the case of nonalignment, *i.e.*, uniform orientation distribution and *c*/*a* ≫ 1 represents molecules highly aligned with the rotor axis.

The experimental ^13^C spinning sideband pattern for tendon has contributions from all the overlapping collagen backbone carbonyl ^13^C and the shielding tensors for these each have an associated orientation with respect to the molecular axis frame. To calculate the spinning sideband pattern for a given molecular alignment, we need to know the orientation of each of the collagen carbonyl ^13^C shielding tensors with respect to the molecular axis frame. There are ∼1000 different carbonyl ^13^C shielding tensors in a collagen molecule and it is clearly impossible to experimentally measure the orientations for all these sites. Instead, we performed QM/MM calculations of the shielding tensors for a (GPO)_7_ collagen-like triple helical peptide (O = hydroxyproline) for which there is a known crystal structure to establish representative shielding tensors, including their orientations, for the primary amino acid components of collagen type I, Gly, Pro and Hyp. QM/MM calculations perform *ab initio* electron structure calculation to yield shielding tensors from atomic coordinates, and from the shielding tensors, the associated ^13^C isotropic chemical shift, chemical shift anisotropy and asymmetry. This approach has been successfully used in the past to calculate ^13^C and ^15^N isotropic and anisotropic chemical shifts in proteins, and benchmark different density functionals for their ability to predict protein chemical shifts.^38^ Here, we performed calculations in Gaussian09^39^ on the central (GPOGPOG)(POGPOGP)(OGPOGP) residues of the (GPO)_7_ triple helix structure, hereafter referred to as “reduced triple helix”, to make the size of the molecular system compatible with the memory requirement of the computer.

The resulting principal values of shielding tensors for the carbons of the central part of the (GPO)_7_ triple helix obtained from our computations were benchmarked against experimental ^13^C CSA measurements performed on a lyophilized (GPO)_12_ peptide^40^ (Figure S3 and Table S1). We found that the calculated isotropic ^13^C chemical shift values and ^13^C asymmetry parameters were in good agreement with the experimental values, whereas the absolute value of the carbonyl ^13^C CSA anisotropy parameter was overestimated by about 10 ppm of overestimation of calculated CSA anisotropies can be expected, because dynamics between possible ground state molecular conformers in the experimental system are not taken into account in the computational model.^38^Figure 4C shows a representation of the calculated shielding tensors of each of the GPO ^13^C nuclei projected onto the triple helix structure, including the tensor orientations (indicated by the orientation of the ellipsoid representing each ^13^C shielding tensor).

### CSA Shielding Tensor Calculations

**Figure 5:**
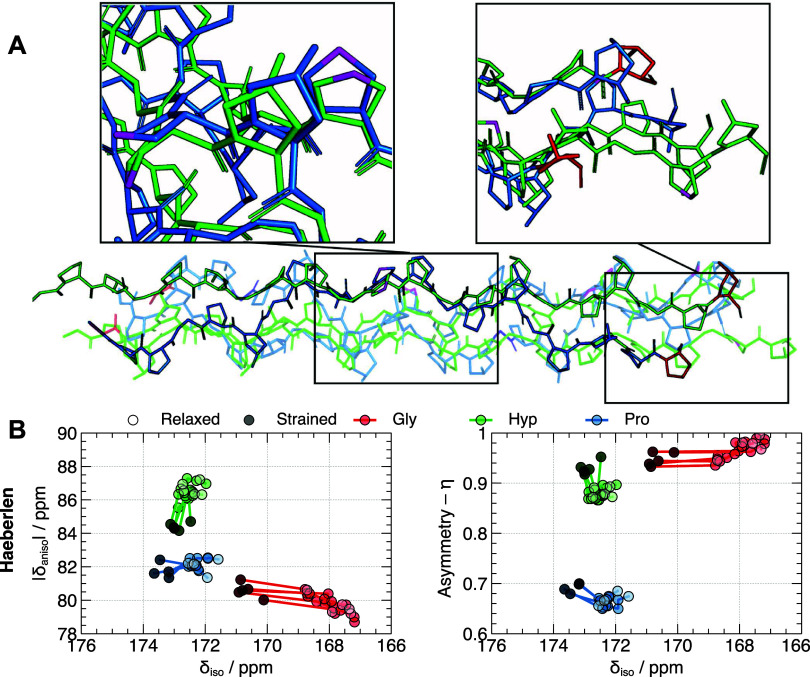
(A) Comparison of the effects of tensile forces on a collagen triple helix: (blue) 10 pN tension resulting in a 1.4% strain and (green) 750 pN tension resulting in a 17% strain. Assuming a collagen triple helix diameter of approximately 1.5 nm, these forces correspond to approximately 5.7 and 424 MPa stress levels. Noteworthy observations include Pro/Hyp C_γ_ residues undergoing endo to exo ring flips (magenta), and extension of the triple helix (red; terminal residues). Additionally, the stretching process appears to result in the increase of the helix pitch. Data for this analysis were sourced from an openly accessible data set from Röder and Rowe.^41^ (B) Calculated ^13^C chemical shift parameters for the C’ in (GPO)_2_ as a function of molecular strain. The initial structure is represented by white circles; progressively applied forces of 0, 10, 50, 100, 250, and 500 pN, result in the chemical shift parameters indicated by black circles. These forces correspond to tensile strains of 0, 1.4, 3.4, 8.6, 13.1, and 17% respectively on the collagen molecules. Note that the same strain applied macroscopically to an intact tissue will not necessarily result in that same strain on individual collagen molecules; strain applied to an intact tissue will move collagen fibrils and molecules relative to each other as well as potentially extend collagen molecules, so the expectation is that larger macroscopic tissue strains would be required to achieve the molecular strains investigated here.

We then sought to gain insight into the trend in changes in collagen backbone carbonyl CSA principal values from changes in collagen conformation between the mechanically strained and relaxed tendon states. We used the (GPO)_7_ structures predicted from MD simulation by Rowe and Röder^41,42^ for strain applied to individual triple helix molecules (Figure 5A) and, as before, we performed QM/MM computations using reduced structures to predict the effect of the strain on the shielding tensors. We calculated the ^13^C shielding tensor principal values, which are plotted against the calculated isotropic chemical shift for each strain value in Figure 5B (Table S3). Figure 5B shows that strain-induced conformational changes in the triple helix are expected to predominantly cause changes in backbone carbonyl isotropic ^13^C chemical shifts, particularly for Gly residues, and conversely, any release of conformational strain would be expected to cause similar but reverse trends in isotropic chemical shifts. In our ^13^C NMR spectra for native tendon (Figure 3A), we see no variation of the carbonyl NMR lineshapes at high MAS rates between the strained and relaxed tendon samples within noise, implying there is a highly similar distribution of carbonyl ^13^C isotropic chemical shifts between the plastic-strain and relaxed states which suggests little difference in collagen molecular conformation between the two strain states.

**Figure 6:**
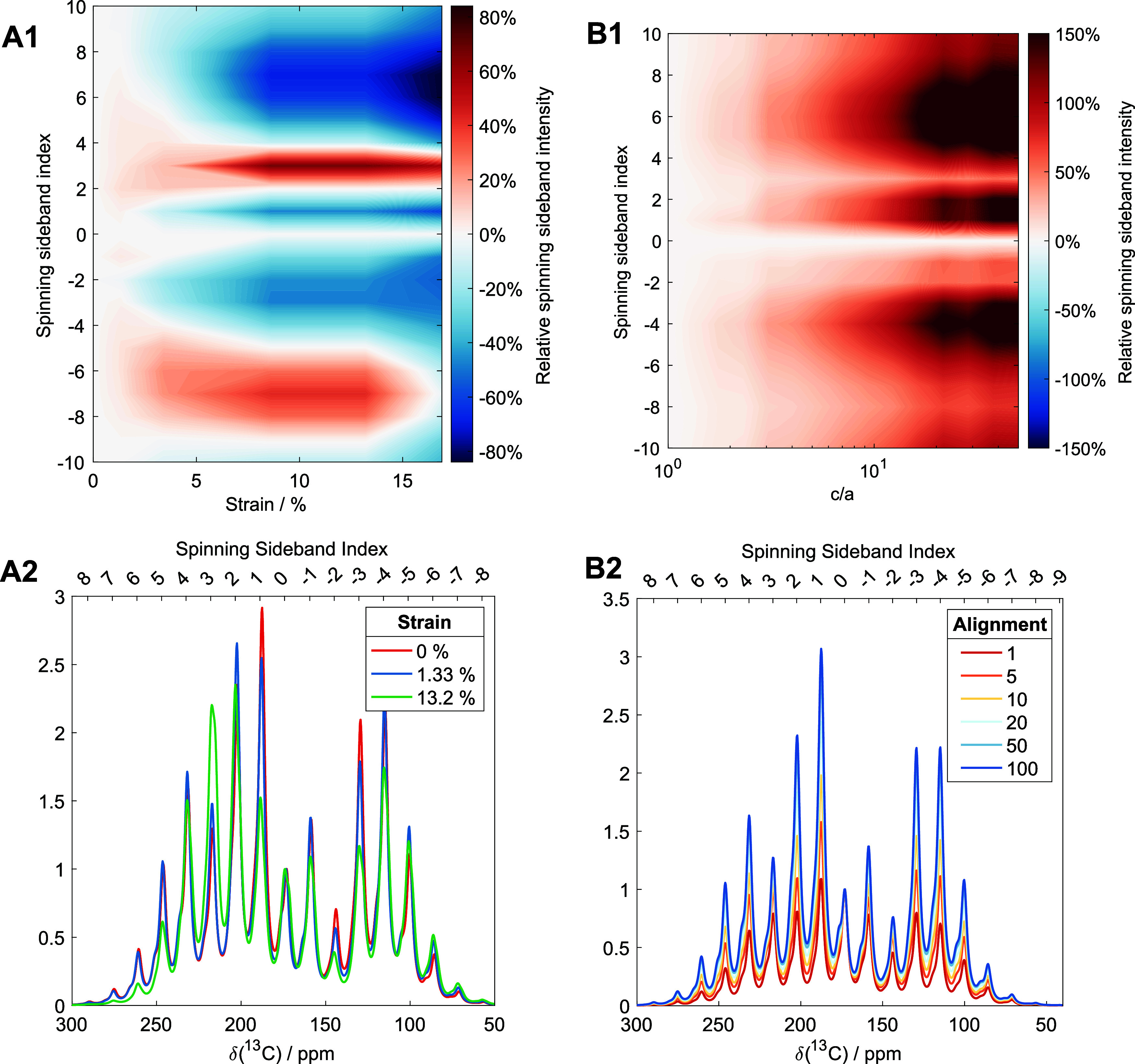
Calculation of 13C carbonyl spinning sideband patterns for strained collagen triple helices for different possible effects of mechanical strain. (A1–2) Effect of changed molecular conformation resulting from strain on collagen triple helices for a fixed degree of molecular ordering (*c*/*a* = 10). (B1–2) Impact of increased molecular ordering or increased preferential alignment of molecules with the NMR rotor axis, defined by *c*/*a* (see Figure 4 for definition). In both cases, the sideband intensities are the sum of those from Gly, Pro and Hyp signals (1:1:1 intensity ratio). Plots (A1, B1) display the total sideband intensity (sum of Gly, Pro, and Hyp signals) as a function of sideband order. In (A1), the sideband intensity is shown relative to a 0% strain condition, while in (B1), the sideband intensity is shown relative to a uniform molecular orientation distribution (where *a*/*c* = 1). Plots at the bottom are simulated sideband patterns. The spectral intensities are normalized on the isotropic chemical shift signal intensity. MAS rate: 2.2 kHz; 14.1 T.

To confirm this hypothesis, we simulated the spinning sideband patterns for the Gly, Pro, Hyp ^13^C carbonyls in the model triple helical GPO peptide using the calculated CSAs above for the *in silico* strained structures for a fixed preferential alignment parameter (*c*/*a* = 10) (Figure 6A1–2). These trends are compared with the spinning sideband patterns that arise from changing the degree of preferential molecular alignment with respect to the rotor axis (defined by *c*/*a*) (Figure 6B1–2). We find that conformational changes from increasing strain result in some sideband intensities increasing while others decrease (Figure 6A1), whereas increasing the degree of preferential molecular alignment results in smooth increase to most sideband intensities relative to the isotropic signal intensity (Figure 6B1). This latter trend is observed in the sideband patterns between the strained (plastic deformation) and relaxed states for our tendon sample (Figure 3). This suggests that the changes in collagen carbonyl spinning sideband intensity patterns between plastically deformed and relaxed tendons come primarily from changes in the degree of molecular alignment, *i.e.*, changes in the degree of molecular order, rather than changes in collagen molecular conformation. As a result, any structural changes to collagen molecules through tensile strain into the plastic deformation region would remain upon release of the strain in the unloaded state. It is possible that conformational changes in collagen molecules from tensile strain are rapidly reversed as collagen intermolecular cross-links break under plastic deformation and the tensile strain on individual collagen molecules is released, or that collagen sacrificial cross-links break before the collagen molecules are strained.^5^

## Conclusions

We have shown that biological structural tissues, here tendon, can be studied under mechanical strain *in situ* by solid state NMR spectroscopy. The method we have developed here for straining mammalian tissues is likely to be equally applicable to mechanical strain studies for other hydrated materials. Even at natural abundance on *ex vivo* tissues, ^13^C CP MAS NMR was sufficiently sensitive to detect subtle changes in collagen molecular ordering. Aided with QM/MM chemical shift calculations and simulations of spinning sideband patterns for different collagen molecular orientation distributions, we find that mechanical relaxation of tendons after strain into the plastic deformation regime results in collagen molecular reordering rather than conformational changes. Collagen molecular disordering could include exposure of cryptic binding sites that allow different integrins to bind to the extracellular matrix. Thus, the chemical extracellular environment that cells detect postdamage may be significantly different to that in pristine undamaged tissue, resulting in a different cell signaling and ultimately altered cell differentiation.

## Materials and Methods

Isolated tails of C57B1/6 control mice, which were not bred specifically for this study, were sourced from the Babraham institute for work approved by the Babraham Institute Animal Welfare and Ethics Review Body, according to the U.K. Animals (Scientific Procedures) Act 1986, license PPL 70/8303. Tendons were extracted as in Stammers et al.^36,36^ Tendons were skinned under pH 7.4 phosphate saline buffer (PBS). One tail has ca. 20 vertebral bones, each of them attached to 4 tendons connecting the bones to the base of the tail. Twisting the tweezers holding the tip caused the tail to break, bringing attached tendon fibers with it attached to the vertebral bone.

For NMR, the tail was broken into two pieces of ca. 10 vertebral bones from the tip of the tail, which resulted in an easier-to-handle bundle of ca. 40 tendons that were attached together at the tip of the tail. Like threading a needle, a thin copper wire loop was passed through a Kel-F hollow tube (OD 2.95 mm, ID ca. 1.9 mm, length 14 mm) that fits snuggly inside a 4 mm rotor, and the tendon were pulled through the tube. Tendons were then strained quickly (2 mm·min^–1^) in a Microtest 200 N tensile stress stage (Deben U.K. Ltd.) fitted with a 200 N load cell. The strain rate minimized the time spent under tension during which cross-links break and physical relaxation occurs. When the plastic deformation regime was reached, as evidenced by the load plateauing at ca. 7%/15 MPa, PBS was quickly syringed out of the Petri dish, and liquid nitrogen was poured on top of the tendon. Only then, the stretching was stopped, parts outside of the inset were cut, and the inset was fitted in a 4 mm rotor kept in liquid nitrogen making sure to drain the rotor to prevent pressure buildup. The tendons were inserted into a precooled probe in the NMR spectrometer typically within 1 min. For the success of this experiment, it is important to ensure that the sample is not allowed to warm up above the water freezing point, which can be followed experimentally by looking for a sharp water line on ^1^H pulse-acquire spectra (compared with Figure S5), which would have been diagnostic of water in some part of the sample melting, which could potentially release the tension on some of the tendons. The probe temperature was set to −60 °C, which corresponded to a sample temperature of −35 °C when spinning.

CSA measurements were performed at 2.2 kHz MAS rate and −60 °C (−35 °C sample temperature), or room temperature and 2 kHz MAS rate for the (GPO)_12_ peptide. CSA fits were performed in the Haeberlen convention^43,44^ using ssNake NMR version 1.4^43^ which fitted the anisotropic chemical shifts, asymmetry parameters, intensity and line widths and generated the static and spinning sideband patterns.

Quantum mechanics molecular mechanics (QM/MM) calculations of ^13^C chemical shift tensors were carried out in Gaussian09 at the b3lyp/tzvp level using the strained collagen structures of Rowe and Röder^42^ as initial inputs.^38^ These calculations incorporated a polarizable continuum model (PCM) to simulate the effects of the surrounding environment, treating the solvent (water) as a continuous medium. This approach allows the influence of the solvent on the chemical shifts to be accounted for without explicitly modeling individual water molecules. To reduce the calculation times, the ends of the triple helices were removed using pdb-tools^45^ (ACE GPO GPO GPO GPO GPO GPO GPO NME)3 → (GPO GPO G)(PO GPO GP)(O GPO GP) and replaced with hydrogen atoms in PyMol (h_add function). The shielding tensors matrices calculated by Gaussian09 were diagonalized to give the principal axis components of the shielding tensors. They were converted into chemical shifts using the equation: δ = −0.9760σ + 175.7662 which was obtained by fitting the isotropic shielding σ_iso_ = 1/3 Tr(σ) to known collagen chemical shifts (Table S2 and Figure S4). The CSA values were calculated in the IUPAC and Haeberlen convention and were visualized in Mathematica after modifying the TensorView script to import and visualize multiple shielding tensors.^46^

Simulations of solid-state NMR spectra under slow MAS conditions (14.1 T, 2.2 or 3 kHz MAS) were performed in Spinach^47^ in the Liouville space formalism. Home-written scripts are available in the Supporting Information. A custom grid was used to simulate the sample alignment, which was generated by probabilistically distributing 800 points on an ellipse, and calculating the Euler angles in the ZYZ convention associated with each point. The axes lengths of the ellipsoid were specified as (10, 1, 1).

## Data Availability

All raw experimental data files, supporting code are available in the Cambridge Research Repository, Apollo, with the identifier: 10.17863/CAM.112392.

## Supporting Information Available

Measured ^13^C T_1_ relaxation times; additional ^13^C CPMAS and ^1^H NMR spectra of the tendon samples used in this work; additional information (figures and tables) on fitting chemical shift parameters to spectra (PDF)
