# Supplementary material for: Solid-State NMR Spectroscopy Investigation of Structural Changes of Mechanically Strained Mouse Tail Tendons
Source: J Am Chem Soc. 2025 Mar 8;147(11):9220–8. doi: 10.1021/jacs.4c13930 (PMC11926861; doi:10.1021/jacs.4c13930)
Supplement: Supplementary file 1 — ja4c13930_si_001.pdf [file ja4c13930_si_001.pdf]

Supplementary information: Solid-state NMR spectroscopy investigation of structural changes of mechanically strained mouse tail tendons

Thomas Kress [1], Melinda J. Duer [1*]
[1] Yusuf Hamied Department of Chemistry, University of Cambridge, Cambridge, United Kingdom
* Corresponding author

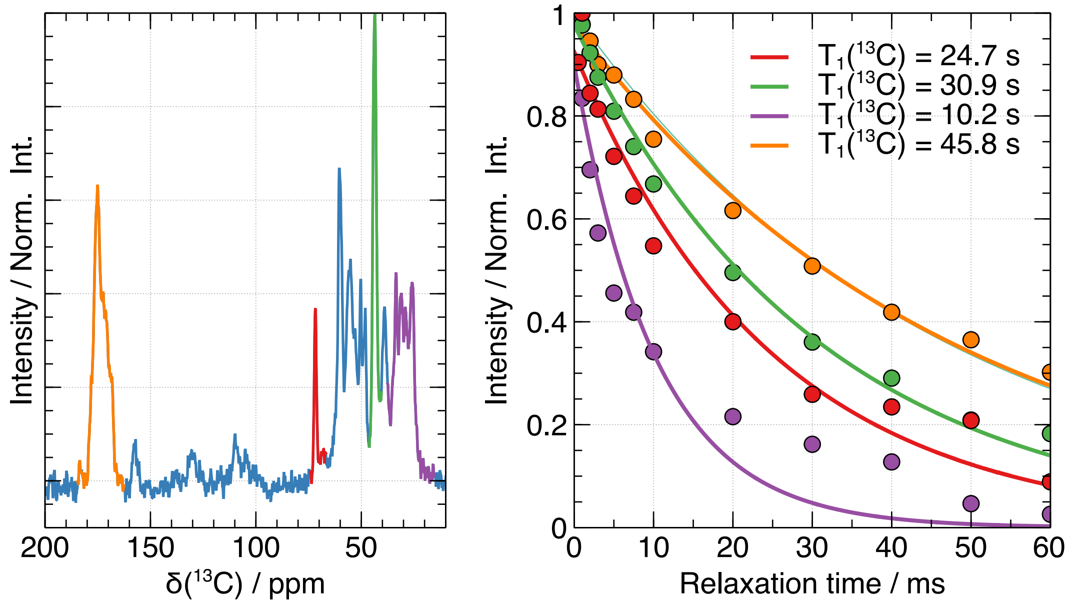

Figure S1: Unstrained mouse tail tendons $T_1(^{13}C)$ relaxation measurements of C', Pro Cg, Gly Ca. The massif of signal between 35 ppm and 10 ppm was fitted with a mono-exponential but might contain multiple superposing components. 14.1 T, 9 kHz MAS rate, -35°C

(A)

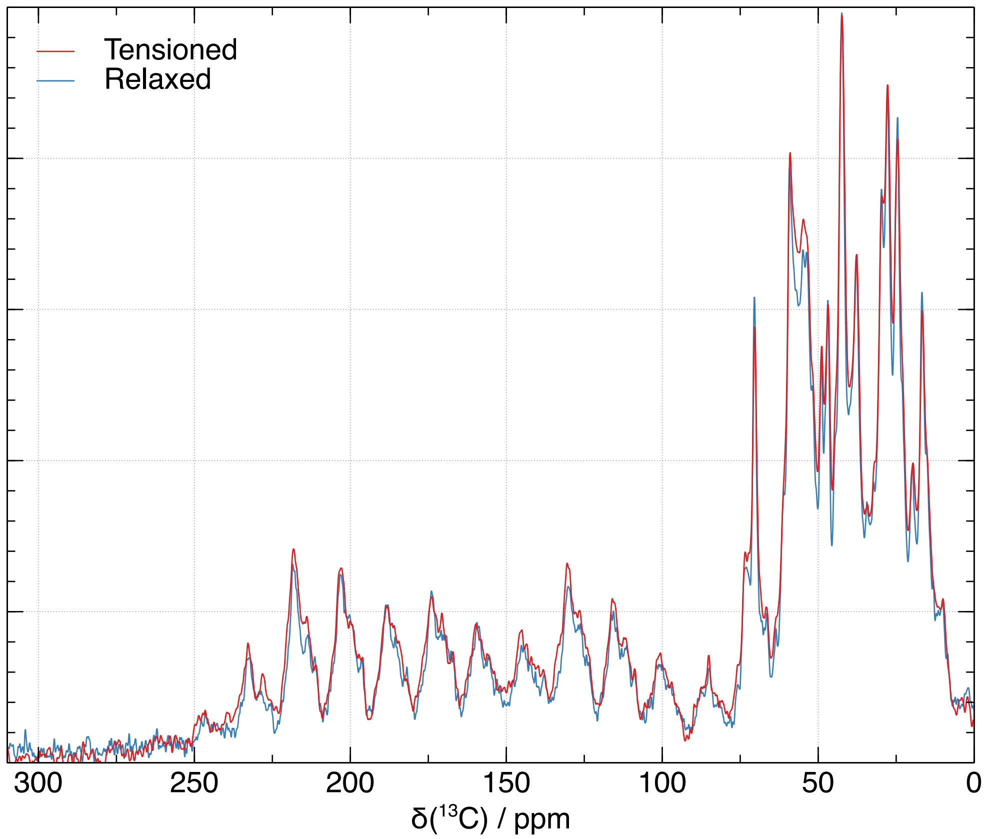

(B)

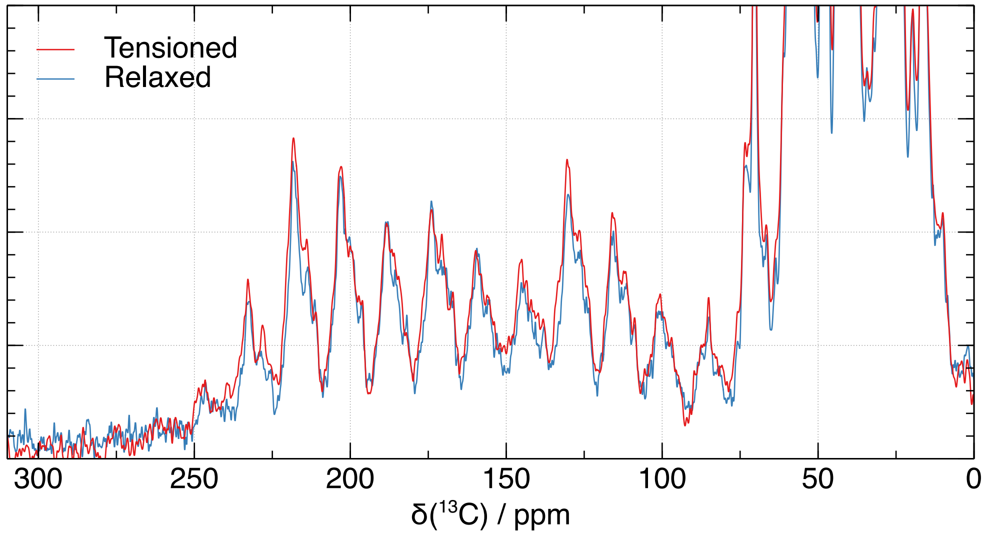

Figure S2 ^{13}C Cross-Polarization (CP) spectra of mouse tail tendons at 14.1T, -35ºC, 2.2 kHz (A) when the spectra are normalized on the intensity of the Gly Cα resonance. (B) when the spectra are normalized relative to the isotropic carbonyl spinning sideband (ca. 172 ppm)

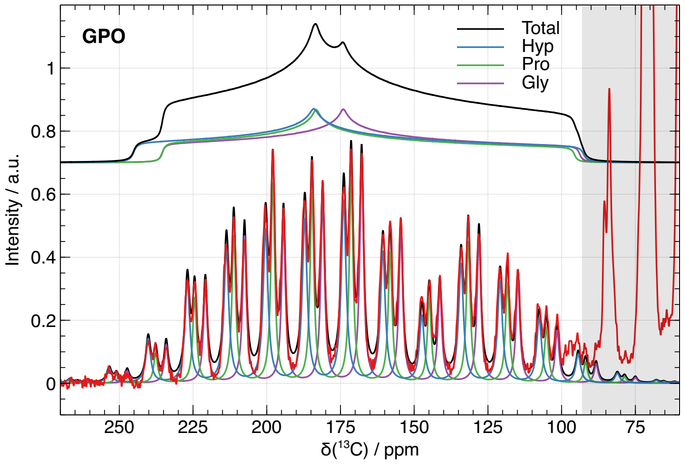

Figure S3 Carbonyl ^{13}C CSA fitting of sideband pattern for (top) a $(GPO)_{12}$ peptide at 2 kHz MAS rate. The grey box indicates regions excluded from the fits, where the spinning sidebands are overlapping with other peaks.

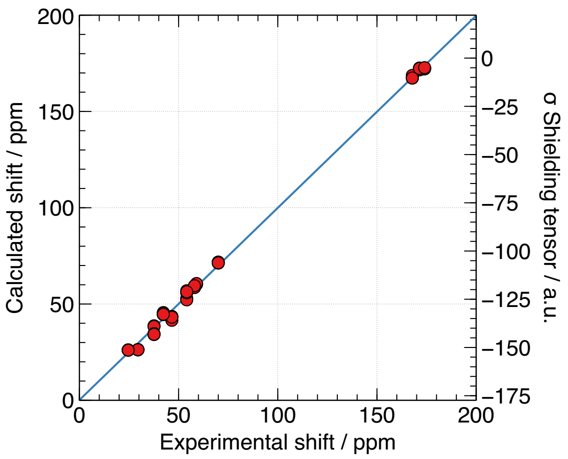

Figure S4 Accuracy of Gaussian09 QM/MM calculations at the b3lyp/tzvp level on a collagen triple helix to calculate the isotropic ^{13}C chemical shifts. These calculations incorporated a polarizable continuum model (PCM) to simulate the effects of the surrounding environment, treating the solvent (water) as a continuous medium. Experimental chemical shifts are given in table S2.

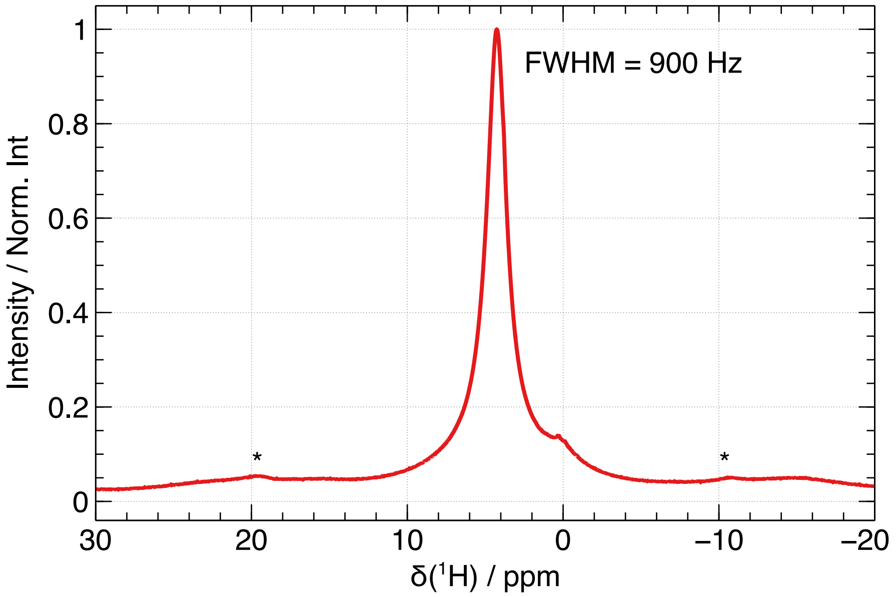

Figure S5 Typical 1H spectra of mouse tail tendons at 14.1T, -35ºC at 9 kHz MAS rate.

Sample	*Assign.*	*Haeberlen convention*			*IUPAC standard convention*			*Linewidth / Hz*
		δ_{iso} / ppm	*δ_{aniso} / ppm*	*η*	*δ_{11} / ppm*	*δ_{22} / ppm*	*δ_{33} / ppm*	
$(GPO)_{12}$	*Hyp*	*173.9*	*-81.4*	*0.8*	*245.6*	*184.3*	*91.97*	*292*
	Pro	*171.4*	*-75.8*	*0.7*	*235.3*	*183.2*	*95.65*	*237*
	Gly	*167.8*	*-73.9*	*0.8*	*235.3*	*174.1*	*93.86*	*243*

Table S1 Carbonyl ^{13}C CSA fitting parameters of control, unaligned tendons and $(GPO)_{12}$ peptide.

	Gly / ppm	Pro / ppm	Hyp / ppm
C	*167*	*171.4*	*174*
Cα	*42.3*	*59*	*58*
Cβ	–	*29.5*	*37.6*
Cγ	–	*24.6*	*71.2*
Cδ	–	*46.6*	*54.6*

Table S2 Isotropic ^{13}C chemical shifts of GPO triple helices used to fit the linear correlation between shielding tensors calculated by Gaussian09, and chemical shifts.

		**Isotropic chemical shift / ppm**	**Asymmetry η**	**|Δδ| / ppm**

**Force / pN**	Strain / %	Gly	Hyp	Pro	Gly	Hyp	Pro	Gly	Hyp	Pro
**0**	0	167.77 ±0.58	172.41 ±0.29	172.11 ±0.43	0.97 ±0.02	0.88 ±0.01	0.67 ±0.01	79.66 ±0.56	86.67 ±0.39	82.05 ±0.48
**10**	1.4	167.82 ±0.58	172.37 ±0.24	172.19 ±0.20	0.98 ±0.02	0.88 ±0.01	0.67 ±0.01	79.59 ±0.66	86.54 ±0.27	82.07 ±0.35
**50**	3.4	168.01 ±0.55	172.54 ±0.20	172.26 ±0.26	0.97 ±0.01	0.88 ±0.01	0.66 ±0.01	79.72 ±0.49	86.55 ±0.40	82.17 ±0.22
**100**	8.6	168.30 ±0.47	172.71 ±0.14	172.41 ±0.11	0.95 ±0.01	0.87 ±0.01	0.66 ±0.01	80.15 ±0.50	86.30 ±0.63	82.29 ±0.25
**250**	13.1	170.66 ±0.33	172.89 ±0.25	173.37 ±0.23	0.95 ±0.01	0.93 ±0.01	0.69 ±0.01	80.59 ±0.43	84.41 ±0.22	81.77 ±0.46
**500**	17	170.66 ±0.33	172.89 ±0.25	173.37 ±0.23	0.95 ±0.01	0.93 ±0.01	0.69 ±0.01	80.59 ±0.43	84.41 ±0.22	81.77 ±0.46

Table S3 Calculated changes of shielding tensors from strained collagen triple helices structures
